# Advanced Morphological and Functional Magnetic Resonance Techniques in Glaucoma

**DOI:** 10.1155/2015/160454

**Published:** 2015-06-08

**Authors:** Rodolfo Mastropasqua, Luca Agnifili, Peter A. Mattei, Massimo Caulo, Vincenzo Fasanella, Riccardo Navarra, Leonardo Mastropasqua, Giorgio Marchini

**Affiliations:** ^1^Ophthalmology Unit, Department of Neurological, Neuropsychological, Morphological and Movement Sciences, University of Verona, 37121 Verona, Italy; ^2^Ophthalmology Clinic, Department of Medicine and Aging Science, G. d'Annunzio University of Chieti-Pescara, 65100 Chieti, Italy; ^3^Department of Neuroscience and Imaging, G. d'Annunzio University of Chieti-Pescara, 65100 Chieti, Italy

## Abstract

Glaucoma is a multifactorial disease that is the leading cause of irreversible blindness. Recent data documented that glaucoma is not limited to the retinal ganglion cells but that it also extends to the posterior visual pathway. The diagnosis is based on the presence of signs of glaucomatous optic neuropathy and consistent functional visual field alterations. Unfortunately these functional alterations often become evident when a significant amount of the nerve fibers that compose the optic nerve has been irreversibly lost. Advanced morphological and functional magnetic resonance (MR) techniques (morphometry, diffusion tensor imaging, arterial spin labeling, and functional connectivity) may provide a means for observing modifications induced by this fiber loss, within the optic nerve and the visual cortex, in an earlier stage. The aim of this systematic review was to determine if the use of these advanced MR techniques could offer the possibility of diagnosing glaucoma at an earlier stage than that currently possible.

## 1. Introduction

Glaucoma is the leading cause of irreversible blindness worldwide. The definition of glaucoma was mainly based on the presence of a typical optic neuropathy along with elevated intraocular pressure (IOP). The IOP still remains the most important risk factor for the onset and progression of the disease. This pathology is characterized by a multifactorial etiology, grouping numerous diseases that share common pathological features of the optic disc [1]. Unfortunately, early diagnosis of glaucoma still remains the main challenge. The literature supports the hypothesis that the first signs of visual field (VF) impairment (that is still the gold standard for diagnosis) appear when a significant amount (between 40 and 50%) of retinal ganglion cells (RGC) has been irreversibly lost [2]. Therefore, diagnostic approaches based on morphology, such as the optical coherence tomography (OCT), have been proposed to anticipate the perimetric diagnosis of glaucoma [3–5]. In the human retina, glaucoma mainly causes the selective death of RGC, whose dendrites comprise the optic nerve, which in turn provides the majority of the input of the primary visual cortex. Previous studies indicated that neuronal plasticity results in a cascade of modifications in structures following modification of neural input [6–8]. Thus a reduction of the number of viable RGC should result in a reduction in the number of dendrites found in the optic nerve, resulting in a decrease in the sensory input of the primary motor cortex, which should decrease the number of functional connections with secondary visual cortex areas. Given that these anatomical structures increase in size with increasing complexity of function, it can be hypothesized that smaller modifications may be more evident in the posterior visual pathway.

Magnetic resonance imaging (MRI) techniques are for the most part nonquantitative; that is, they are not directly comparable over time. Techniques that do provide quantitative results include morphological measurements such as cortical thickness measured on anatomical scans, diffusion tensor imaging (DTI) that provides information concerning the microstructural integrity of white fiber tracts, arterial spin labeling (ASL) that provides a quantitative estimate of blood perfusion in mL of blood per mg of tissue, and functional connectivity (FC) that estimates the synchronization of anatomically distinct regions of the cortex from blood-oxygen-level-dependent contrast imaging (BOLD) acquisitions. The aim of this systematic review was to determine if these advanced MR techniques could possibly diagnose glaucoma at an earlier stage than that which is currently possible with the VF.

## 2. Methods Used for Literature Searches

PubMed searches were performed on July 31, 2014, using the phrases “glaucoma” and “primary visual cortex” and either “freesurfer” or “voxel-based,” “optic nerve” and “diffusion tensor,” “primary visual cortex” and “arterial spin labeling,” and “primary visual cortex” and “functional connectivity” for publications from 1980 to April 2014. Articles in English were fully reviewed. Articles in other languages were reviewed only when an abstract in English was available.

## 3. Results

### 3.1. Morphometry

Techniques to quantitatively evaluate morphological aspects of the brain can be divided into two principle groups: voxel-based and surface-based. The first uses a statistical approach to assign a probability that a voxel is occupied by grey matter. The latter determines vertices that define the surface of the grey matter-cerebral-spinal fluid and the grey-white matter interfaces and uses these vertices to estimate the cortical thickness with a submillimeter precision.

Bogorodzki et al., in a study on the voxel-based technique [9], reported that a group of 14 patients with unilateral vision loss due to end-stage open-angle glaucoma showed a statistically significant thinning of the visual cortex compared to a group of 12 normal age-matched subjects.

In another study with advanced glaucoma patients that used a 3-dimensional magnetization-prepared rapid gradient-echo sequence of MRI and optimized voxel-based morphometry a significant decrease in the bilateral grey matter volume in the lingual, calcarine, postcentral, superior frontal, and inferior frontal gyrus and rolandic operculum as well as in the right cuneus, right inferior occipital gyrus, left paracentral lobule, and right supramarginal gyrus was found [10].

Hernowo et al. [11] found a significant reduction in volume of the visual pathway structures between the eyes and the visual cortex, including the optic nerves, the optic chiasm, the optic tracts, the LGN, and the optic radiations, in eight patients with VF loss due to primary open-angle glaucoma (POAG). Based on these findings, the authors proposed that MRI, in combination with automated morphometry, could be used to aid the detection and assessment of glaucomatous damage posterior visual pathways.

Li et al. [12] reported that atrophy and degeneration of the visual-related cortex existed in the dorsal and ventral visual pathways in the advanced stage of POAG but were absent in the early stage.

Interestingly, Williams et al. reported that brain changes varied according to the glaucoma stage. The right and left inferior occipital gyri, right middle occipital gyrus, right inferior temporal gyrus, and right occipital lobe white matter were larger in glaucomatous patients than in healthy subjects. In multivariate regression analysis, 38% of all brain structures had independent associations between decreasing volume and more advanced glaucoma. These findings suggested that in patients with glaucoma the extent of cortical brain changes correlates with disease severity [13].

Given that the cortical thickness of the visual cortex showed a strong correlation with aging, when corrected for sex [14], one may speculate that a possible diagnostic test could be the comparison of a patient suspected of having glaucoma in an early stage to a group of healthy controls with the same age. Or the patient with manifest glaucoma could undergo repeated MRI scans in order to identify modifications in cortical thickness induced by disease progression.

**Figure 1:**
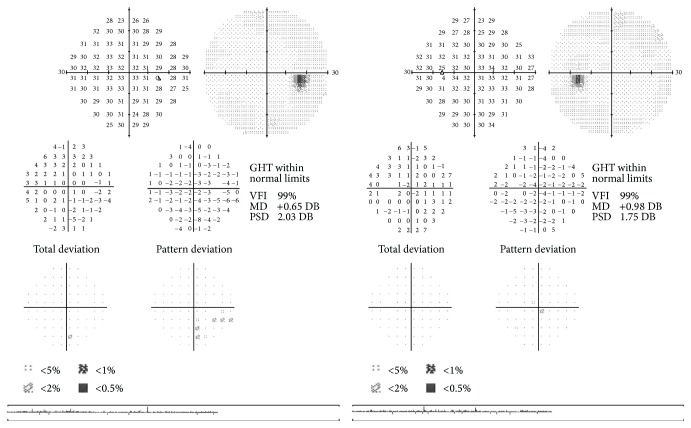
Normal visual field examination (Humphrey field analyzer II 750 (Carl Zeiss Meditec, Inc., Dublin, CA; 30-2 test, full-threshold)) of a patient in an early stage of glaucoma.

**Figure 2:**
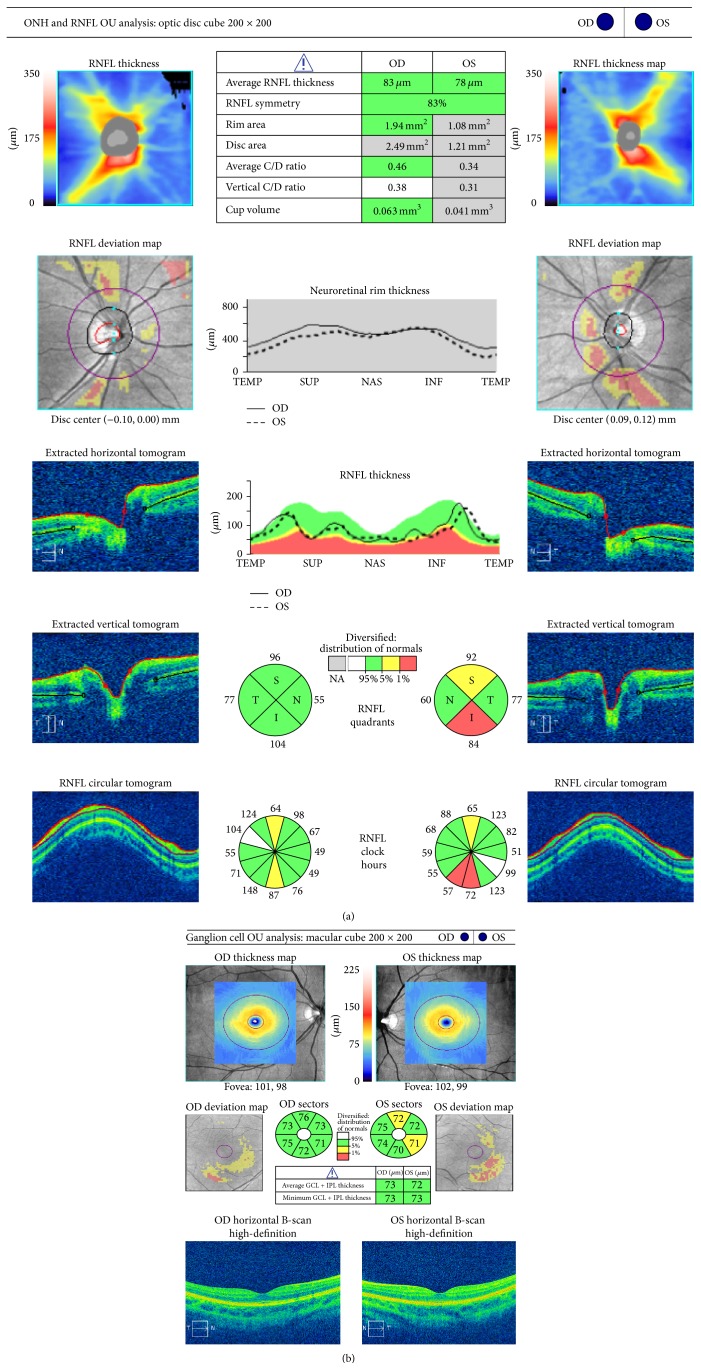
Spectral domain optical coherence tomography (Cirrus, Carl Zeiss Meditec, Inc., Dublin, CA, version 6.5 software) of the same patient, showing the peripapillary RNFL (a) and macular GCL/IPL (ganglion cell layer/inner plexiform layer) (b) glaucomatous defects.

**Figure 3:**
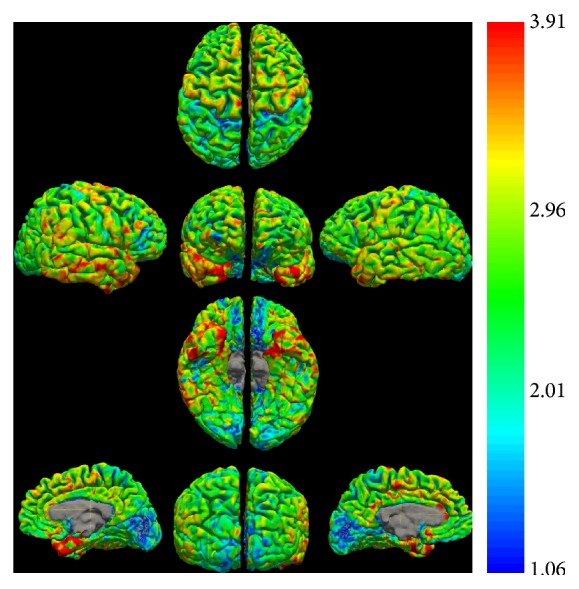
Cortical thickness estimates of the same patient as Figures 1 and 2. Note that the major concentration of the thinnest cortex (blue on the color scale) is located in the primary visual cortex and presents a greater extension on the left hemisphere.

Figures 1 to 3 represent a patient with early stage glaucoma. While VF is still normal (Figure 1), morphological defects of the retinal nerve fiber layer (RNFL) (Figure 2(a)) and ganglion cell layer/inner plexiform layer (GCL/IPL) complex (Figure 2(b)) (as seen with spectral domain optical coherence tomography) are already evident. MRI morphological technique for evaluating cortical thickness shows a thinned cortex in the primary visual area (Figure 3).

### 3.2. Diffusion Tensor Imaging

**Figure 4:**
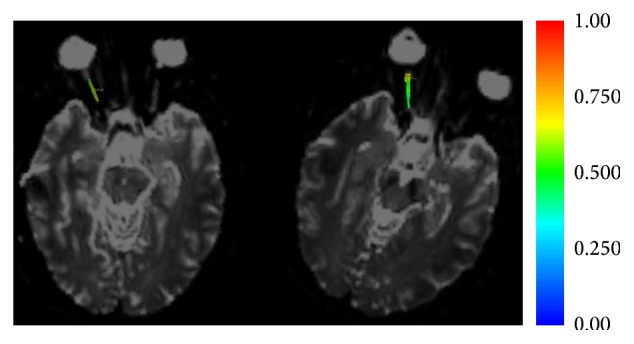
DTI of the same patient as Figures 1 and 2. The fibers traced on the right optic nerve are colored with the fractional anisotropy measured at each point along the nerve (see legend). Note that the variation in color on the right (classic isometric sequence) is greater than that on the left (a priori sequence with the majority of the directions lying in the direction of the optic nerve). The greater variation indicates a greater variability in measurement: in other words the standard deviation of the sequence on the right is greater. The higher precision of the new sequence would be useful for the smaller modifications that should be present in the early stages of glaucoma.

DTI noninvasively evaluates the microstructural integrity of tissue by sensitizing the MRI signal to the random motion of water molecules via diffusion encoding gradients. Tissue that is organized as fibers, such as nerves and white matter tracks, gives a highly anisotropic signal. A modification towards a more isotropic signal indicates a loss of structural integrity.  Figure 4 shows two DTI scans using classical acquisition protocol on the right and a priori information concerning the orientation of the optic nerve on the left in the same patient shown in the previous figures (Figure 4). The latter shows an improvement in signal-to-noise ratio, a more homogenous measurement, and an increase in length of the traceable fiber lengths.

Xu et al. [15] evaluated the use of this imaging modality to investigate the optic nerve in mice and humans. They reported that the optic nerve degeneration following retinal ischemia exhibited a distinct pathological progression. The axonal injury preceded demyelination without significant destruction of overall axonal cytoskeletons in their mouse model. These modifications could be detected with parallel and perpendicular diffusivity, respectively. The same results were not observed in humans probably due to technical difficulties.

Fiedorowicz et al. [16] reported that changes observable with DTI in the optic nerve correlated with the progression of glaucoma. Specifically, they observed a statistically significant correlation between the increase in mean diffusivity (MD) and a decrease in fractional anisotropy (FA) with increasing severity of glaucoma.

Michelson et al. [17] reported that the morphology of the papilla correlated, after elimination of the effect of covariates, with the axonal integrity and demyelination of the optic radiation in controls and glaucoma.

Garaci et al. [18] evaluated both the optic nerve and the optic radiation and reported that these structures had significantly higher MD and significantly lower FA in patients with glaucoma compared to controls. Notably, MD and FA in the optic nerves correlated with glaucoma severity, suggesting that these parameters could serve as complementary indicators of disease severity.

Nucci et al. [19] reported that MD and FA were correlated with morphological features of the optic nerve head and retinal nerve fiber layer (RNFL) documented with scanning laser polarimetry (GDx-VCC), confocal scanning laser ophthalmoscopy (Heidelberg Retina Tomograph, HRT-III), and optical coherence tomography (Stratus OCT). Specifically, MD displayed the strongest correlation with linear cup/disc ratio (LCDR) from HTR-III, RNFL thickness from OCT, and nerve fiber index (NFI) from GDx, while FA strongly correlated with LCDR. These findings suggested that DTI could be a valuable complementary diagnostic method to assess structural modifications of retinal ganglion cells and optic nerve in glaucoma.

In another study by the same group [20], the authors found that at early glaucoma stage MD values were higher at the proximal site of the optic nerve head with respect to the distal site. On the contrary, a decrease in FA was observed only relative to patient stage, independent of optic nerve site. Moreover, during the early stage of glaucoma, an increase in overall diffusivities was evident at the proximal site, whereas at the distal site a decrease of the largest diffusivity and an increase in both the intermediate and the smallest diffusivities were observed. The authors concluded that the high sensitivity of FA along with the high specificity of MD at the proximal site could provide reliable indexes for the identification of structural damage at early stages.

The main limitation is the diameter of the human optic nerve, which is on the order of 3-4 mm and is located in an area that shows magnetic field susceptibility artifacts [21]. This limit can be overcome with the use of modified diffusion tensor techniques and with the improvement of the signal-to-noise ratio obtainable with newer and higher magnetic field MR scanners.

### 3.3. Arterial Spin Labeling

ASL measures tissue perfusion by labeling the water present in blood (i.e., modifying the orientation of the spins) in one volume and then measuring the amount of these spins after the blood has passed into another volume. The measurements of perfusion are comparable with those obtained with positron emission tomography [22].

Lavery et al. [23] reported that a reduced ocular blood flow in DBA/2J mice compared with C57BL/6 control mice. This result supported the hypothesis that ischemia or hypoxia was a possible contributing factor in the optic neuropathy in the DBA/2J mouse model of glaucoma.

Duncan et al. [24] reported that resting blood perfusion in human primary visual cortex in 10 patients with POAG correlated with the loss of visual function. They concluded that altered cerebral perfusion was an indication of postretinal glaucomatous neurodegeneration caused by damage to the retinal ganglion cells.

Presently, the main limitations of ASL are the lack of its widespread diffusion and the low signal-to-noise ratio that increase the acquisition time. These limits will probably be overcome with the increased diffusion of high field scanners and improved acquisition protocols and postprocessing techniques.

### 3.4. Functional Connectivity

Resting-state FC is the connectivity between brain regions that share functional properties. After the acquisition of a BOLD sequence of a patient at rest with their eyes open, postprocessing determines which regions present a temporal correlation in BOLD-signal fluctuations. In order to evaluate the visual system or any other functional system, a predetermined set of regions of interest are used (seed based). To date, only two studies have been published using this method to evaluate the visual system.

Dai et al. [25] reported that the FC of the visual cortex with associative visual areas in 22 patients with POAG was modified compared to 22 age-matched healthy controls. There was also a disrupted connectivity between the primary and higher visual areas. That is, the communication between the primary visual area and higher visual cortices was decreased, as was the positive FC between both of these areas and more remote regions of the brain. The authors speculated that areas of increased positive FC with visual cortices may represent compensatory recruitment or diminished inhibitory input.

Frezzotti et al. [26] reported abnormalities in structure and FC within and outside visual system in 13 patients with advanced POAG compared to 12 age-matched healthy controls. These modifications correlated with VF parameters in poorer performing eyes. These findings suggest that structural and functional changes in glaucoma go beyond the visual system, suggesting that POAG can be considered a neurodegenerative vision disease falling within the group of neurodegenerative disorders and, as such, results in widespread modifications of the brain.

The main limitations of FC analysis include difficulties and time required for postprocessing of the BOLD acquisitions and interpretation of the results.

## 4. Summary and Conclusions

Glaucoma is a multifactorial disease that involves retinal ganglion cells and also structures of the central visual pathway [8]. The application of progressively more advanced imaging technologies in the field of glaucoma is rapidly growing, allowing a more accurate knowledge of the pathophysiology of the disease, earlier diagnosis, and a better evaluation of responses to therapies [5, 27, 28].

The introduction of advanced neuroimaging techniques may facilitate the study of the entire visual pathway, opening new frontiers in the early detection of the disease and in the evaluation of the therapeutic efficacy of novel neuroprotective strategies.

In summary, voxel-based morphometry can detect modifications of visual pathways structures mainly in advanced stages of glaucoma. Even though morphometry shows an evident correlation between cortical brain modifications and the stage of disease, the application of this method for the early diagnosis of the disease still has limits. DTI generally found an increase in MD and a decrease in FA in patients with glaucoma, modifications that significantly correlated with increasing severity of disease. Interestingly, these modifications also occurred at early stage of glaucoma, being more evident at the proximal site of the optic nerve head with respect to distal sites. This suggests that each portion of the optic pathway should be precisely pondered when attempting to identify the initial signs of disease. Studies that used ASL to detect cerebral perfusion abnormalities in patients with glaucoma are very limited. Presently, the application of this method has focused on identifying contributing factors involved in optic neuropathy onset and progression. FC documented important alterations between primary and associative visual areas and also between nonvisual areas, suggesting that glaucoma should be considered as a neurodegenerative disorder with widespread ramifications in the brain.

In conclusion, the number of studies that evaluated modifications observable with advanced MRI techniques is progressively growing even though especially limited in patients with glaucoma in advanced stage. But the results support the potential for these techniques for detecting the modifications induced in an early stage of glaucoma.
